# *Onlife* Extremism: Dynamic Integration of Digital and Physical Spaces in Radicalization

**DOI:** 10.3389/fpsyg.2020.00524

**Published:** 2020-03-24

**Authors:** Daniele Valentini, Anna Maria Lorusso, Achim Stephan

**Affiliations:** ^1^Institute of Cognitive Science, Osnabrück University, Osnabrück, Germany; ^2^Department of Philosophy and Communication, Bologna University, Bologna, Italy

**Keywords:** radicalization, algorithms, internet, onlife, media, Islamic State

## Abstract

This article argues that one should consider online and offline radicalization in an integrated way. Occasionally, the design of some counter-measure initiatives treats the internet and the “real” world as two separate and independent realms. New information communication technologies (ICTs) allow extremists to fuse digital and physical settings. As a result, our research contends that radicalization takes place in *onlife* spaces: hybrid environments that incorporate elements from individuals’ online and offline experiences. This study substantiates this claim, and it examines how algorithms structure information on social media by tracking users’ online and offline activities. Then, it analyzes how the Islamic State promoted *onlife* radicalization. We focus on how the Islamic State used Telegram, specific media techniques, and videos to connect the Web to the territories it controlled in Syria. Ultimately, the article contributes to the recalibration of the current debate on the relationship between online and offline radicalization on a theoretical level and suggests, on a practical level, potential counter measures.

## Introduction

The police stop a young adult at Bologna’s airport with downloaded Islamic State (ISIS) propaganda on his phone. Upon the detainee’s release, he reaches out to the Italian branch of the radical platform *al-Mohajiroun*, and he is subsequently re-routed to London where the network holds its headquarters. After prolonged online contact, and a real-world friendship with his future co-conspirators, he decides to act; he kills 8 people during the so-called London Bridge attack. This description pictures the radicalization of Youssef Zaghba, a Moroccan-Italian terrorist who died in 2017. To what extent did he develop violent tendencies while socializing in cafés and parks? What role did digital chatrooms, and their contents, play in funneling his radicalization?

Radicalization is a contested concept with some definitional loopholes. But in this paper, we will stick to the growing consensus among scholars who consider it as a process of developing extremist beliefs and ideas while condoning the use of violence as legitimate (McCauley and Moskalenko, 2008). The exponential growth of new information communication technologies (ICTs) has prompted experts to consider their impact on terrorist activity. Subsequently, the internet has become the hallmark of modern radicalization patterns. Whether it is the easiness of signing up to homogeneous radical communities, unobstructed access to a deluge of violent footage, or the possibility to reach a global audience, the Web has become a hotbed for terrorist recruitment. However, offline bonds and social circles exert a massive, and sometimes crucial, influence on people’s violent leanings, too (Weimann, 2015; Winter, 2015).

Research recognizes the importance of questions that enquire how, and where, radicalization takes place. However, the analysis of physical and digital relations in radicalization has also yielded conflicting results and unproductive countermeasures. Multiple authors have suggested that unsatisfactory results may be connected to a dearth of empirical data and to the formation of a “false dichotomy” that views online engagements as separate from physical relations (Ducol, 2015). This paper will follow their steps: it will argue that radicalization is better conceived as a process that unfolds online, and offline, simultaneously in a hybrid *onlife* space, to use an expression coined by Floridi (2015). This *onlife* space seamlessly integrates elements that pertain to both the online and offline spheres.

Before moving on, we think it is useful to delineate at the outset some theoretical and methodological aspects that define the scope of the present study. On the one hand, the *onlife* approach we adopt is to be considered as the latest stage of a research branch that has and keeps calling for the integration of digital and real-world features in detailing out both radicalization patterns and possible countermeasures (section Online vs. Offline Radicalization: A “False Dichotomy”; Wojcieszak, 2009, 2010; Scrivens et al., 2019). On the other hand, we will delve into these problems focusing on a particular kind of terrorism-related radicalization: ISIS. We consider this case particularly interesting for the ways in which ISIS has set its radicalization practices in-between online and offline experience, thus providing a benchmark for the analysis of the contemporary strategic attunement between these two dimensions. Moreover, even though we single out different radicalization contexts in which an *onlife* framework may yield fruitful results – and other authors have recently applied it to examine lone actor cases (Fisogni, 2019) – the specifics we take into account for ISIS’ *onlife* radicalization cannot automatically be generalized to other radicalization forms. In fact, the aim of the present study is mostly theoretical: we want to outline, through the case studies analysis, some helpful concepts and attributes to better understand the *onlife* character of ISIS’ radicalization strategy. Further research is needed to see, if and how, our contribution may be extended to other radicalization contexts.

In methodological terms, we adopt a qualitative approach for the analysis of our corpus. We will carry out a textual analysis of a selection of ISIS recruitment videos, looking at the narrative values they shape and at the mechanisms they use to merge the reality and the textual level. Following a textual-semiotic approach, we consider texts (in our case videos) not to be pure representations of something, but actors intervening in complex processes that confer sense to the world we live in and to the actions we perform. This is exactly why it is important to look at texts: they give us behavior models, shape our beliefs, and offer an image of us which, very easily, influences our actions. Any text, according to the semiotic approach (Lorusso, 2015; Walsh Matthews, 2017), works in this way, but this mechanism of “return-effect” is even more evident in the *onlife* dimension of social media. Similar to the current standard in textual analysis, our corpus has no statistical relevance. We choose these texts because they seem particularly “dense” and significant, to underline some typical discursive mechanisms of the dynamic online-offline relationship in today’s radicalization. Furthermore, they can also provide guidelines for the construction of corpuses in other radicalization contexts. Ultimately, we think that an *onlife* conception of radicalization can help counter-terrorism specialists develop tailored strategies to curb the appeal of extremist groups and terrorist organizations.

In the following we proceed as follows: in the first section, we problematize the separation between online and offline radicalization by first reviewing previous studies that caution policymakers and experts against the ineffectiveness of such separation (Turpin-Petrosino, 2002; Bliuc et al., 2019); then we use recent empirical results, which show that the distinction is a false dichotomy (Neumann, 2013; Gill et al., 2017). In the second section, we consider how algorithmic data-gathering activity that manages digital communications on social media platforms benefits from users’ previous online history and offline interactions. We examine how algorithms structure radicalization-oriented echo-systems that merge virtual habits with offline features. In such *onlife* environments users complete their radicalization process surrounded by like-minded associates and consensual media footage. In the third section we use the *onlife* framework to describe ISIS’ radicalization strategies: ISIS provided its potential recruits with interacting physical and virtual environments (caliphates) that substantiated its own *us* vs. *them* ideology. Here, “caliphate” refers to the swath of land that ISIS controls (physical caliphate), and to the internet spaces that ISIS exploits to broadcast its state-building project (digital caliphate) (Atwan, 2015). Scholars described the capability of cross-cutting the events happening in both the digital and the physical caliphates as one of the distinctive features at the basis of ISIS’ radicalization success (Winter, 2015, 2018). We argue that the intersection of a physical entity and a digital cognate stresses the *onlife* character of ISIS’ recruitment strategy. This, however, must be considered as an ISIS-specific maneuver that may have a poor application range. In fact, to the best of our knowledge, no other terrorist organization or extremist movement – be it right-wing, left-wing or religious – has geared its members toward violence using two co-sustaining spaces in the same way ISIS did. Nevertheless, sociopsychological studies and recent reports highlight that far-right movements manipulate the concept of homeland and create white-only online habitats along ISIS lines (Mols and Jetten, 2014; Conway et al., 2019) but their operations are not the same as the *onlife* state-building project of ISIS. The fourth section considers how services offered by instant-messaging applications replicate, and reinforce, the affiliative dynamics that underlie the radicalization of small, isolated cliques. Specifically, we examine how Telegram’s patrolled chatrooms, and encrypted secret chats, offer extremists protected locations to foster their radicalization. The final section uses a representative narrative corpus – in the sense mentioned above – to analyze how ISIS has been able to conjugate the *onlife* environment by implementing a multitude of reality-like effects that are scattered throughout its videos.

## Online Vs. Offline Radicalization: a “False Dichotomy”

The so-called Web 2.0^1^ and further versions, and new ICTs, have been game-changers in radicalization’s layout, but their effects do not restrict to radicalization patterns. It is evident that these technologies have deeply changed many forms of our life in general, and of recruitment in particular (from politics to social works). Similar to most companies, terrorist organizations took advantage of internet-based affordances, and they moved a great swath of their operations to the online world (Amble, 2012; Awan, 2017). Recruitment, propaganda, network-building, financing, and logistics entered the virtual arena with such a strength that King and Taylor point out that terrorism cases without a *digital footprint* have become rare (King and Taylor, 2011). As the internet’s mark on radicalization processes gained traction, scholars started to debate the impact of digital environments on such processes. Some argued that consuming jihadi videos on a frequent basis exerts a facilitative effect by motivating individuals to engage in violent action (Holt et al., 2015). Others, in contrast, envisaged the internet merely as an accelerator of radicalization, but did not credit the platform with an essential role in the process (Van der Valk and Wagenaar, 2010). More recently and mostly separately, literature started to discuss radicalized individuals’ relationship with both cyber interactions and with face-to-face interactions (Archetti, 2015; Klausen, 2015). While there is a growing consensus that radicalization comprises both kinds of relations, research has yielded mixed results as to the extent in which online interactions, and their physical counterparts, are interlocked in the pathways of radicalized people (Weimann, 2012; Pauwels and Schils, 2016). Scrivens et al. (2019) list a series of limitations of and provide suggestions about how the study of the internet’s impact on violent extremism should progress. Among the issues they call out, two are of particular interest for our scope: first, a scarcity of primary data that facilitates a lack of evidence; second, the necessity of “drawing connections between the on- and offline worlds of violent extremists” (p. 3) and, thus, of avoiding the implementation of ineffective countermeasures developed along a false dichotomy “which artificially distinguishes cyberspace from the ‘real world”’ (Ducol, 2015, p. 90).

The scarcity of primary data has, of course, to do with high-risk security issues that would force experts to stay in war zones and to encounter dangerous individuals (Silke, 2004). As a result, most studies that concern the extremist use of the internet are not empirically based; an issue that affects terrorism studies in general (Sageman, 2014). With a few excellent exceptions (von Behr et al., 2013; Gill and Corner, 2015; Koehler, 2015; Gill et al., 2017), the vast majority of research – including this one – relies on secondary data and anecdotal episodes that are usually gathered from newspaper articles and other gray literature (Ducol, 2015). In so doing, the type and quality of data prevents researchers’ rigorous examinations of the internet’s influence in radicalization’s promotion. This limitation is irrespective of whether violent exposure, or the conduits of cyber-interactions, propel the process. Conway (2017) details future investigation avenues, and remarks that the study of internet-based radicalization has been hindered by a dearth of data-driven descriptive and explanatory research. She suggests that “basic descriptive research is largely missing from this field, along with more complex theory-informed approaches” that seek “to show causal connections” (p. 78).

The other critical issue is the missing acknowledgment of the reciprocal influence that online associations, and physical bonds, bear on radicalization. Sometimes, when analyzing the trajectories of different radicalized offenders “scholars tend to conceptualize virtual spaces as autonomous from what actually happens in the “real world” and vice versa” (Ducol, 2015, p. 90; Gill et al., 2017). As a relic of Web 1.0, where the boundaries between static websites, and people’s flesh-and-bone interactions were more clearly defined (Jenkins, 2006), the conception of the digital and the physical sphere as two fully-encased spaces gives a misaligned representation of the mechanisms involved in radicalization. The widespread use of such approaches in counter radicalization programs is surprising, especially if we consider that the intimate codependence between digital interactions and their possible offline spillovers has been well-established in research branches strictly connected to terrorism studies like internet and communication studies (Conroy et al., 2012). For example, Carolyn Turpin-Petrosino examined the responses of teenagers and university students to the exposure to hate groups’ propaganda and their attitude toward the latter (Turpin-Petrosino, 2002). After word-of-mouth and phone contact, internet was the third most successful technique in provoking a change of attitude among users. However, 20 years later, both word-of mouth and phone calls have been incorporated into the digital world. Crucially, as we will see in section two, algorithm-based pieces of technology have created a frictionless relationship between conversations and actions happening in the real word and those taking place online. The connection between on- and offline bonds has been more explicitly investigated in a series of self-report studies by Magdalena Wojcieszak. The author underlined how both similar and dissimilar offline social ties exacerbate the ideological extremism of Neo-nazis and radical environmentalists participating in online forums (Wojcieszak, 2009, 2010). Many respondents reported that encountering diverging opinions offline made them delve even deeper into their extremist ideology with the aim of elaborating suitable counterarguments. As a result, she advised that engaging extremists with alternative perspectives might have detrimental counter-terrorism effects. Not only did her findings extend those of previous studies about the bearing of both online and offline interpersonal discussion on political civic engagement (Hardy and Scheufele, 2005; Shah et al., 2005); they were also confirmed by recent survey and longitudinal studies focusing, respectively, on the link between e-participation and a variety of offline pro-active activities (Tai et al., 2019) and on the repercussion that local riots have on the Australian white supremacy online community in terms collective beliefs, emotions and consensus (Bliuc et al., 2019).

These insights notwithstanding, we can observe the magnitude that the on-and-offline false dichotomy has enjoyed among violent extremism experts. On a theoretical level, the dichotomous conception has led Sageman (2008a, b) to argue that “[d]uring the past two or three years, however, face-to-face radicalization has been replaced by online radicalization. The same support and validation that young people used to derive from their offline peer groups are now found in online forums, which promote the image of the terrorist hero, link users to the online social movement, give them guidance, and instruct them in tactics” (p. 41). Likewise, Omotoyinbo (2014) outlines that “radicalization, in the age of ICT, is basically of two ramifications i.e., Offline and Online” (p. 54). On a practical level, the same reasoning has produced some questionable one-sided countermeasures, such as the FBI *Don’t be a Puppet* or the campaign *Think Again Turn Away*. The former initiative, in its address of potentially radical individuals, excluded the impact of offline interactions altogether. The latter campaign was concerned only with fighting ISIS’s online presence, and it did not consider its offline side-effects in stigmatizing Muslim communities in the USA (Davies et al., 2016).

Counter-terrorism experts must consider how social interactions in today’s world incorporate elements that pertain to digital artifacts and to people in “real” social settings. In addition, the border between the Web and the physical world becomes fuzzier and fuzzier (Burrows, 2009; Dunbar et al., 2015). As we will explore in the next section, social networking sites, coupled with mobile devices, enable meanings, beliefs, and emotions to be concomitantly experienced in the two spheres to the point that it becomes hard to tell where the individual ends and the user begins (Floridi, 2015). Take for example the Christchurch mosque shootings in March 2019. Australian lone actor Brenton Terrant entered the building and murdered 50 people while recording his brutal attack on a head-mounted camera and broadcasted it on Facebook. In doing so, not only did he “air” a terrorist attack online that, unfortunately, inspired some copycats; his misdeed was the concrete performance of his pre-attack post that claimed how it was “time to stop shitposting and time to make a real life effort post” (Conway et al., 2019, p. 14). As the example shows, in modern extremism, the internet and physical spaces conflate in unprecedented ways. However, one should keep in mind that, the *onlife* degree in different radicalization cases – the hybridization between online and offline settings – is to be intended along a continuum. In some cases, digital and physical interactions interlock in such a way to maximize the radicalization’s *onlife* magnitude, whereas in others, the role of virtual and “real-life” components is more discernible.

All in all, both theoretical and empirical studies call for a reconsideration of such a dichotomy. In terrorism research, Lohlker (2011) investigated the relationship between the internet and Al Qaeda operatives’ radicalization strategies. They concluded that the aim of internet jihadis is “to make the divide between the virtual and the physical more permeable with the help of elaborate media strategies. The participants in discussions call more and more for the keyboard to be exchanged with the detonator” (p. 9–10). And this is precisely what Humam al Balawi did on December 30, 2009 when he blew himself up and claimed the life of CIA agents. Informative is the fact that in his last essay Balawi rhetorically stated: “when will my words drink from my blood!?” (in Lohlker, 2011, p. 1 3). His dreadful actions confirmed the second hypothesis of the study according to which “virtual activity creates real terrorist” (p. 64). Along similar lines, Peter Neumann cautions against the implementation of one-sided countermeasures like content removals. Such actions, for example, would be unproductive in the long run given the vast amount of platforms on which contents can be disseminated, and, most importantly, they would deprive intelligence services from gathering useful information on terrorist behavior. On the other hand, he emphasized the link between virtual and physical radicalization by predicting that terrorist organizations will carry out their radicalization project by conjugating the material portability of smartphones and the digital character of phone apps (Neumann, 2013; see section Physical Entitative Groups and Encrypted Online Networks). More recently, Gill et al. (2017), in their study that analyzes the use of the internet among 223 UK convicts on terrorism charges, are very explicit about the risks connected to the applicability of the above-mentioned dichotomy. They ultimately conclude:

“There is no easy offline versus online violent radicalization dichotomy to be drawn. It may be a false dichotomy. Plotters regularly engage in activities in both domains. Often their behaviors are compartmentalized across these two domains. For example, plotters may engage in face-to-face interaction regarding the ideological legitimacy of their actions while engaging in virtual communication regarding the technical specificity of bomb-making” (Gill et al., 2017, p. 114).

The operationalization of this divide seems to stand on a slippery slope even in episodes of lone-actor terrorism. Lone-actors are defined as isolated individuals who develop an affinity for radical ideas and violent tendencies in the seclusion of their accommodations, and who avert any sort of group membership or external contact. This is why their presence and terrorist plots are so hard to anticipate and disrupt (Hoffman, 2003; Spaaij, 2010). A cursory glance at the previous definition and case studies – like Anders Breviek who murdered 77 civilians in Norway, 2011 – grants some leeway to the impression that this perpetrator typology acts solely on its own. However, a deeper inspection reveals that this is seldom the case. In a recent study, Lindekilde et al. (2019) contend that, often, these supposedly under-the-radar actors display their “leakage behavior” through the establishment, and maintenance, of interpersonal bonds with leaders, peers and, sometimes, co-conspirators. The magnitude of these relationships (or alternatively the degree of loneness) might change on a situational basis: pockets of individuals may engage only in intermittent peripheral contact, while others may showcase a higher degree of embeddedness within extremist circles. Ultimately, the contention that lone-actors decide to embark on solo terrorist missions should not divert researchers’ considerations of the impact that outer relations have on lone-actors’ radicalization processes (Malthaner and Waldmann, 2014). Importantly, such bonds are both virtual and physical. Physical and digital connections in lone-actors are so intertwined that “online and offline patterns on radicalization often occur simultaneously and are mutually reinforcing, and exclusive online radicalization of isolated individuals is exceedingly rare” (Lindekilde et al., 2019, p. 5). Schuurman and colleagues based their investigation on similar conclusions, and they labeled lone-actors as “the typology that should not have been” (Schuurman et al., 2018, p. 771). Once again, contrary to popular belief, their article values the impact of virtual and physical radical milieus on lone-actors’ motivation, and capacity, to carry out a terrorist attack. These findings echo Neumann and Steven’s previous research, which, despite acknowledging the new radicalization potentials that are ascribed to cyberspace, continue to highlight the unquestionable influence of real-world ties in self-radicalization instances (Neumann and Stevens, 2009). Koehler provides further evidence that justifies the dismissal of this dichotomy. In his interviews, German Neonazi’s answers suggest how the online, and the offline, dimensions feedback-loop into one another in terms of ideology buttressing, propaganda dissemination, and rally participation (Koehler, 2015).

While further in-depth analysis is needed to understand the extent of cyber-interactions in the promotion of politically violent acts, we will introduce a further “algorithmic” add-on as to why counter-terrorism experts should cautiously avoid such online/offline divide in the design, and implementation, of counter-narrative strategies.

## Algorithms and Dataveillance: an *Onlife* Meaning-Making Mechanism

In this section, we argue that a technological reason as to why the offline vs. online dichotomy is due for an overhaul dwells in the systems that regulate online interactions: algorithms. Radicalization studies have allocated little attention to the principles that govern algorithmic data-gathering activity in the structure of radicalization-friendly environments. This sounds surprising, given that the US National Security Agency claimed to have nipped more than fifty terrorist attacks in the buds through the extraction of data from social media (Van Dijck, 2014). In light of media philosopher Marshall McLuhan’s lesson, who already over 50 years ago declared that the medium is the message (McLuhan, 1964), we intend to show how radicalization that unfolds through social media interactions is partially constituted by the software and codes that make up the medium (Burrows, 2009). Crucially, algorithms draw intensely on user’s offline resources in their predictive performance, and they render online radicalization a more “physical” or “offline” experience than it is usually thought (Cohen, 2018).

Taken at face value, we tend to picture algorithm-based systems as autonomous, efficient, platforms that carve the contours of our virtual scenery (Finn, 2017). They instantly present us with information that satisfies our search queries, interests, and desires. In addition, algorithms’ operations under the surface of users’ online experiences reinforce the “illusion of platform neutrality” (Gillespie, 2010; Milan, 2015, p. 3): the information that we receive on our Facebook newsfeed looks as objective as it can be. But, far from being neutral and objective, social media algorithms come inscribed with a series of biases that skew the content selection process; they determine “what there is to know and how to know it” (Bozdag, 2013; Gillespie, 2014, p. 167). Put differently, they are information-filtering systems that are preset with specific ideological proclivities and design choices, and they prime certain features while neglecting others (Finn, 2017). For instance, when algorithms scaffold the media ecology on a user’s laptop, they feed on a vast amount of signals that encompass previous online history, recently contacted users, and social gestures, i.e., likes and comments (Bozdag, 2013; Gerlitz and Helmond, 2013). However, the accumulation of so-called “dataveillance” is just as important: that is, tracked information that refers to users’ offline habits. This information includes location, shopping purchases, and phone calls (Degli Esposti, 2014; Van Dijck, 2014). A final important, and yet often undermined, aspect that shapes the algorithmic environment is the collection of negative media data, i.e., time spent away from the platform, or typed in – but unposted – comments (Cohen, 2018).

When all of the abovementioned ingredients are taken into account, it becomes clear that one can regard algorithm-run systems as complex, sociotechnical artifacts that interlock human-machine interactions in a continuous process of content production. In other words, these calculating vehicles represent a sophisticated instantiation of the “dynamic cognitive flows between human, animal, and machine” that constitutes the *cognisphere* that we live in Hayles (2006, p. 165). Algorithms are adaptable systems that co-evolve along with their users. Their filtering mechanisms aim to structure a mediascape that is responsive to the updated data-based profile of each user so as to maximize the time spent surfing the platform (Cheney-Lippold, 2011). Indeed, one congenital goal of social networking sites is the creation of engagement. People who usually succeed in persuading others to prolong, or resume, their online interactions – by initializing a soon-to-become viral thread for example – are deemed as soft leaders. However, this mediatic “participation by default” (Gerlitz and Helmond, 2013, p. 14) is hardly an all-algorithmic business. On the one hand, it is true that a portion of the user’s digital dossier is built with data “collected passively without much effort or even awareness on the part of those being recorded” (Meyer-Schoenberger and Cukier, 2013, p. 101). On the other hand, the building of someone’s digital image (datafication) requires a great amount of physical labor. A user’s geolocation, network nodes’ activity (friends), and user-curated information influence recommendations. In other words, the user explicitly provides metadata and tags that allow the algorithmic mechanism to shape the information it receives. For example, if I am looking for a restaurant or a car, I am much more likely to get a diner that is nearby my current position, or a car that belongs to a close friend of mine – provided that she posts a car-selling advertisement (Bozdag, 2013). The resultant outcome of this filtering operation is an immersive environment that is tailored to meet users’ past, present, and anticipated tastes. Such computational customization directs users toward a personalized online experience that is equipped with deeply ingrained relational traits. Algorithms cherry-pick contents while scanning our everyday social spheres in order to present us with a vast hodgepodge of “entry points” to stay hooked up to the platform (Willson, 2017). Furthermore, algorithms’ *modus operandi* is sustained by the diffusion of portable devices: laptops and mobile phones provisioned with apps such as Facebook and Whatsapp. These apps allow algorithms to continuously structure an up-to-date datafied image of individuals. Data, in fact, might be partial and incomplete, but they are anything but raw materials (Gitelman, 2013). As soon as they are collected, data undergo a refinement progression that is purposed to design a sufficiently fine-grained user profile. This refinement progression keeps the latter entangled in the virtual infrastructure.

Social media platforms are dependent on these algorithmic systems, and they have been said to act as polarizing tools that promote exposure to pro-attitudinal contents, and easy contact, with digital like-minded networks (Dylko et al., 2017). In other words, experts maintain that new media threaten to create homogenous digital echo chambers. Individuals inhabit these chambers where only in-group consonant outputs are circulated at alternative views’ expense (Sunstein, 2017). The level of algorithmic interference in the creation of echo chambers has been questioned, however. Large-scale studies measuring the impact of algorithm-suggested news on selective exposure and polarization highlight how users’ choices are more influential than machine-run activities in the creation of echo chambers (Bakshy et al., 2015; Boxell et al., 2017; Beam et al., 2018). Even though we agree that environmental bias should not be overemphasized in the construction of secluded online spaces, we simultaneously stress how the context in which interactions occur should not be overlooked^2^. Here we side up with Steglich (2019) when he claims that “to blame the negative side effects of […] echo chambers on individuals’ decisions, and downplay the role of the algorithms […] is a flawed, incomplete and dangerous conclusion. These individual decisions take place in a highly pre-structured environment [which] pre-determines the […] outcome of the decisions” (p. 22). For instance, simulation studies analyzing the effects of friendship recommender systems on social media found that these platforms promote a frequent network rewiring that may lead to the creation of isolated social triads (Sasahara et al., 2019). If such triads are inhabited by radicalizing individuals, then social media algorithms could be seen as a partial contributor of violent extremism. In other words, when it comes to radicalization, a selective exposure apparatus regulates extremism-orientated online echo chambers that encase “at risk” individuals. These echo chambers are safe heavens, where violent intents – surrounded by large amounts of radical narratives – are developed and embraced (Sunstein, 2002; Atwan, 2015; Maggioni and Magri, 2015). It is true that social media companies have curbed the building and expansion of extremist echo chambers through frequent account and content removal (Berger and Morgan, 2015). Nevertheless, the multiplatform nature of the internet safeguards their survival and continuation. What is more, counter-terrorism strategists’ efforts may fall on deaf ears under the very mechanisms that govern algorithmic activity. For one thing, if further information on a user’s screen is based on frequently consumed content, counter-messages may never enter the mediascape of potential recruits in the first place. For another thing, even if counter-terrorism storylines “hit” their target audience, their alleged purpose might backfire. Individuals, in fact, appropriate meanings in accordance with the position that they occupy in a specific social network that is both online and offline, and during their radicalization, potential recruits usually lurk in hardline networks (Archetti, 2015). For example, the US government campaign *Think Again, Turn Away* aired in 2013, which aimed at discouraging ISIS supporters from migrating to Syria, has proved to be counter-productive; among other things, it fantasized the Caliphate as a nightmarish place of destruction. Jihadi supporters were advised not to buy a “return ticket,” since they would have found only bombings and death there. In a nutshell, the campaign championed the high probability of death as a root cause to stay home. But inadvertently, uncompromising Salafist youths considered the very same death dimension on online social settings as the only way to reach the bliss of martyrdom (Katz, 2014; Van Eerten et al., 2017).

While we subscribe to the influence of online echo chambers on radicalization, we propose that online echo chambers are better considered as echo-systems that incorporate digital, and real-world, elements alike in light of the algorithms’ filtering mechanism. Importantly, contents and interactions conducive to radicalization intersect artifacts, environments, and bodies in a dynamic fashion as the algorithms and the individuals seamlessly feedback loop information into each other. An ISIS French video, which involves stabbings and decapitations, in a user’s recommendation list might be the combined outcome of her online consummation of similar footage in the past, her offline life in France, and her purchase of a knife a couple of days before. Admittedly, the algorithm has access to the knife purchase’s information if it is made with a credit card that is connected to an online bank account. In doing so, the algorithms may register such a purchase, and it may match it with knife-related tags in the video. Moreover, mobile devices can allow me to watch, and comment, on such videos, while I am simultaneously engaged in a physical meeting with other peers.

Here and in the following, we follow the lines of Luciano Floridi and colleagues when we argue that (radicalized) individuals should be better regarded as individuals who populate an *onlife* infosphere: a new dimension that characterizes human beings in the contemporary algorithm-based era (Floridi, 2015). Floridi contends that, in this third space, “the digital is spilling over into the analog and merging with it” in unprecedented, and sometimes unforeseeable, ways (Floridi, 2007, p. 62), and suggests that “the threshold between *here (analog, carbon based, off-line)* and *there (digital, silicon-based, online)* is fast becoming blurred” (ibid. p. 63; italics in the original). *Onlife* interactions are creating *connected information organisms (inforgs)*, and they are resorting to digital and physical artifacts to go by with their lives. In our view, algorithms and portable devices are just the latest manifestation of the continuous interactive dynamics between online, and offline, dimensions. Another advantageous feature of the *onlife* dimension that could explain why terrorist groups have been so fond of the Web 2.0 is the “shift from the primacy of entities to the primacy of interaction” (Floridi, 2015, p. 63). In other words, people in this hyperconnected era establish their identities and beliefs by leveraging on multiple relationships that fluctuate primarily from *onlife* intimate groups (family, peers) and, subsequently, expand into the larger society (Floridi, 2015, p. 98). This conception fits well in the relational approaches to radicalization, whereby the process takes place “in a dynamic constellation of multiple spaces and social relationships over time” (Lindekilde et al., 2019, p. 5). Algorithms make the digital and physical settings all the more intertwined. In the next section, we will analyze how ISIS managed to intertwine the online, and offline, sphere in its recruitment process.

## The *Onlife* ISIS Recruitment: *Us* vs. *Them*

The Islamic State has been proclaimed defunct. After a concerted military effort that lasted about 4 years, a coalition of more than sixty countries managed to quarantine this once proto-state to a handful of in-land outposts. However, what now resembles an insurgent group has been the latest uncontested protagonist of the jihadi galaxy. Over the past 5 years, an unprecedented wave of foreign fighters replenished its militia manpower, thus securing the possibility to first conquer, and later administer, a territory as big as the United Kingdom. Figures suggest that 30,000 conscripts voluntarily flocked to ISIS-controlled Syria to partake in its utopian governance project; 5,000 conscripts were of Western descent (Schmid and Tinnes, 2015). So-called returnees’ recent terrorist attacks in Paris and Brussels, with a combined death toll of 132 civilians, showcase these conscripts’ continued security threat. As a result, governments invested massive funds to stop this extremist human hemorrhage, and academic circles started to peruse the root causes of ISIS’s appeal (Milton, 2016). A dissection of Islamic State’s paraphernalia of narratives, which it weaves to lure young recruits to the so-called Caliphate, shows a distinctive feature that stitches such propaganda together. This feature concerns the presence of an overarching enemy that assails the Islamic identity (Schmid, 2015; Gartenstein-Ross et al., 2016). This ideological position is nothing new: literature that concerns social movements and intractable conflicts is replete with examples of radical groups that feel engaged in a Manichean struggle against an evil enemy, and the Islamic State is no exception (McCauley and Moskalenko, 2011; Della Porta, 2013; Halperin, 2016). From a theoretical standpoint, ISIS inherited al Qaeda’s well-established ideological template and it brushed this template with convenient theological interpretations (Schmid, 2014). Indeed, Mark Sedgwick points out that the modern “jihadist account of the existing order” posits “a fundamental division between Muslims and non-Muslims, and that Muslims are suffering because of non-Muslims” (Sedgwick, 2012, p. 368). The paramount narrative of *ummah*, the imagined global community of Muslims that should be re-united under an Islamic banner away from illegitimate powers, compounds this inter-religious division (Cook, 2005; Campanini, 2008). To be a viable, and practical, concept in jihadist circles, the *ummah* presupposes the complementary existence of a non-Muslim adversarial conglomerate that has split Islamic devotees apart since time immemorial (Günther, 2014). Consequently, the Islamic State portrays itself as the bastion of the ultimate faithful that confronts the aggressive attacks of “infidels”. Its manifesto encourages infidels’ annihilation to secure the unadulterated continuation of the whole Muslim community. This ideological operation amounts to a black-and-white worldview granted with an *us* vs. *them* perspective where the presence of the enemy embodies an existential threat (McCants, 2015; Stern and Berger, 2015).

Burgeoning evidence suggests that adherence to such a binary and emotionally charged worldview is one of the main levels that pushes enlisters to increase the ranks of radical violent organizations (Horgan, 2014; Bronner, 2016). Relatedly, the Muslim/non-Muslim divide molds a cognitively inflexible plateau of unequivocal boundaries that is prone to stir potential recruits toward radicalization (Hogg et al., 2013). ISIS enforces this simplistic separation, and it eviscerates the potentially inconsistent motivational salience that comes from all the other socially relevant categories – gender, age, nationality, educational level, and occupation – in the name of a dogmatic, and easily applicable, religious congruity. However, if an intransigent *us* vs. *them* ideology is all it takes to persuade thousands of violent Salafists toward radicalization, this would not explain how, and why, al Qaeda – the jihadist organization *par excellence* – failed to mobilize such a critical mass. What really sets ISIS apart from its competitors is the Caliphate’s unexpected announcement; a step that Bin Laden and his associates never ventured to take.

The revival of this highly revered religious-political entity as the righteous land for the Muslims allowed the 2014-branded Islamic State to experience an exception inflation of recruits. But why is that so? Surely, the Caliphate – in the pious Muslim mindset – is connected to a regime of sacred values and temporal apocalypticism that social psychologists and sociologists might engender as the acceptance of violence as a political opinion amongst hard-liners (Atran et al., 2014; Berger, 2015; Roy, 2017; Winter, 2018). Core to the present study, the proclamation of the Caliphate substantiated the *us* vs. *them ideology* with a spatial dimension that blended virtual and physical interactions. For example, in ISIS’ online-magazine Dabiq (2014), al-Baghdadi proclaimed a physical proto-state whose vicissitudes and shape were tightly coupled with its digital counterpart. He thereby fueled an *onlife* radicalization process. ISIS, in fact, was quick to set foot in the digital arena: they carved out cross-platform spaces where its potential recruits could partake in its own constitution by joining the cyber-army of sympathizers and proselytizers who shared and celebrated ISIS’s war victories. This is not to say that there is no distinction between online and offline. It suffices to say that the physical Caliphate’s borders are now non-existent, and the living conditions in war-flagged Syria have no resemblance with the image that is fabricated by ISIS propagandists. Nonetheless, as far as recruitment goes, the making of the actual Islamic State, as the in-group physical institution, is feedback-looped into a virtual duplicate that is populated by jihadi comrades. Indeed, Islamic State is also a multi-platform digital Caliphate where radicalization could begin and continue; it seems driven by the mutual interdependence of these two spaces (Price et al., 2014; Atwan, 2015).

Put differently, on the internet, “at risk” individuals, fostered by algorithmic mechanisms, could safely inhabit growing radicalization echo chambers that were directly connected to on-the-ground developments. Indeed, it is no exaggeration to say that ISIS *onlife* state-building project enjoyed disproportionate media coverage up to the point that some experts claimed it to be “the sole source of its appeal” (Winter, 2018, p.106). For example, studies suggest that the direct online engagements between foot soldiers that broadcasted battle segments on social media, and male users who watched and messaged them, was a paramount component in the radicalization process’s escalation (Winter, 2015). Winter considers this point, and he argues how these enlisters represent the living embodiment of the actual jihadist who tips potential recruits over the edge by bridging the distance between the bedroom and the battlefield in a manner that propaganda alone simply cannot. The Twitter campaign mounted by ISIS around the death of Muath al-Kaseasbeth is another instance of how meanings, maneuvers, and beliefs crossed physical and digital settings. Before the Jordanian Pilot’s execution, the Islamic State launched an online survey that asked its cyber militants for the most suitable capital punishment (Griffin, 2014). The hashtag #weallwanttoslaughtermoaz went viral in jihadist online circles, and it made online members’ abilities to “have a say” in the execution process possible. Likewise, women were attracted to migrate to the Caliphate by the perspective of becoming the founding mothers and wives of a Sharia-ruled land. Inspired by communication with and pictures of women employed as nurses, teachers or police forces, hundreds of Western Muslim women flocked to Syria to live out “their religion in a congenial environment” (Peresin, 2015, p. 24). A prominent ISIS female recruiter known as Umm Ubaydah wrote that for her, as well as for others, a core reason to move to Syria was the willingness “to build an Islamic State that lives and abides by the law of Allah” (Hoyle et al., 2015, p. 12). The former description highlights the *onlife* character of ISIS’ radicalization strategy. In other words, radicalized individuals could swarm an expanding digital Caliphate: an extended online environment that provided a space for individuals to do battle with the enemy, and it also presented individuals with a foretaste of expectations in Syria by partaking in the construction, and dissemination, of physical developments (Fisher, 2015). It is no coincidence, in fact, that ISIS’s territorial extension, and the number of foreign fighters, goes hand-in-hand with its digital media capability and presence (Berger and Morgan, 2015; Nanninga, 2019).

At the end of this section, we will sketch some suggestions that future research may take as prompts to extend the *onlife* framework that we have outlined to the analysis of far-right movements. However, before doing so, we put forward some elements that make ISIS’ *onlife* strategy ISIS-only and, thus, limit the scope of our analysis. First, we have to consider the very state-building project: ISIS managed to militarily seize and control a physical territory and ruled over it with a religious iron fist. The broadcasting of the chance to join armed battles and to implement laws and regulations is something that no other extremist organization – whether right-wing, left-wing or jihadi – can grant their members with. A clue that supports the importance of this aspect is the incredible amount of “air time” that ISIS propagandists devoted to war and victory media outputs, during its peak (Winter, 2015). Second, in spite of the contested role that the institution of the Caliphate played in the course of Islamic history, the latter is still revered by Muslims from across the West and the Middle East alike. For instance, the popularity for the resurgence of the Caliphate is made clear by a 2007 poll result from four major Muslim countries that revealed that sixty-five percent of respondents wished to live under a single Sharia-based country (Pankhurst, 2013). Beside the survey, Islamic historian Wael Hallaq penned down a detailed analysis about the differences between the modern European state and the conception of state based on Islamic sources and declared the impossibility of reconciling the two (Hallaq, 2013). His analysis provides keen insights for the examination of why the Caliphate might be highly praised by a segment of Islamic population. ISIS, on its part, seems to have taken these considerations into account and riddled its messages with powerful Caliphate-related and religious narratives that have remained surprisingly stable along the years (Kuznar, 2017).

Over the last few years, the far-right scene has developed an *onlife* radicalization project that, in some respects, resembles the tactics employed by ISIS. We notice how these movements follow the lines of violent Islamism and use specific media strategies to transfer the battleground in front of the users. Just like ISIS invited potential recruits to share battles online to later join ISIS physically, right-wing inspired lone actors stream their terrorist acts to let similarly minded individuals participate and copy what they do (the Halle synagogue shooting was inspired by the Christchurch massacre and both were available online). Furthermore, sociological and socio-psychological research has proved how extreme right-wing movements and leaders mobilize their members by evoking the narrative of an ethnically homogeneous homeland that is easily replicable online (Mols and Jetten, 2014). A growing amount of evidence, in fact, underlines how right-wing groups use internet platforms to churn out white supremacy-only spaces (Conway et al., 2019). At first glance, these *onlife* maneuvers around the notion of homeland come close to the ways in which ISIS uses the concept of Caliphate in its radicalization project. However, a blunt comparison between the two would amount to an inappropriate interpretative stretching. In fact, right-wing groups neither have a swath of land where to implement their worldview, nor is the concept of homeland deeply-ingrained in mainstream European society. Notwithstanding these differences, we envisage multiple avenues for the comparison of ISIS and far-right groups *onlife* strategies (Al-Rawi, 2018; Schwarzenegger and Wagner, 2018). One is the topic of the next section: how encrypted messaging services facilitate the *onlife* radicalization of small cliques.

## Physical *Entitative* Groups and Encrypted Online Networks

We borrow Donatella della Porta’s words when we say that ideological encapsulation and militant enclosure are two recurring features of the radicalization process (Della Porta, 2013). Ideological encapsulation is the radical individuals’ acceptance of blunt *us* vs. *them* reasoning. Moreover, militant enclosure signals how such individuals often mature violent leanings while they socialize in small affiliative cliques. In short, it is no exaggeration to say that radicalization is about who you know (Dalgaard-Nielsen, 2010). Malthaner’s recent study analyzes the formation of the so-called Sauerland-Group, a terror cell plotting attacks in Germany. In this study, he concludes that “the group emerged from a radical network that formed within the wider *Salafist* movement and to which it remained connected during preparations for violent attacks” (Malthaner, 2014, p. 648). However, while scholars have recognized the significance of broader social networks as a receptacle of new recruits, they have also noticed that those among extremist circles progressively sever their ties with their surroundings, and they continue the radicalization in a more isolated and intimate location. This process is usually referred to as “going underground” (Della Porta, 2013; Malthaner and Waldmann, 2014; Decety et al., 2018). Social psychologist Michael Hogg suggests that the physical segregation of micro-cliques tends to produce entitative groups: closed units of individuals that are endowed with clear boundaries, internal homogeneity, and a well-defined inner structure where black-and-white ideologies, and the adoption of aggressive actions, are allowed to breed on a fertile ground (Hogg, 2012).

In fact, these insular entities of like-minded people not only provide terrorist cells with a hiding place away from the surveillance of intelligence services; they are also likely to take the radicalization process to an extreme extent through the conjoined conduits of specific cognitive, and affective, dynamics. Research has shown that exclusive social interaction in such self-confined spaces may augment the divide between in-group and out-group members up to the point that the latter get completely deprived of their humanity. These out-group members may become the worth target of harmful actions (Waytz and Epley, 2012; Kteily et al., 2016). In the case of ISIS, this dehumanization operation may possess even greater magnitude in consideration of the explicit raw treatment that they give to their victims in countless gruesome videos. In addition, entitative groups display an equivalent predilection toward in-group members. The restricted socializing setting ensures a perceived similarity among different individuals that culminates in the reciprocal development of strong affective attachments and congruent cognitive interpretations.

Recent technological advancements have found a way to substantiate similar digital dynamics, and they have opened up new avenues for private, and public, isolated communication. The use of encrypted messaging services, like Whatsapp and Telegram, allows individuals to easily exchange private texts, and other media products, in safe environments. These platforms can engage peer-to-peer, and closed group, communication without any content being leaked to undesired users or third parties. Ultimately, it appears that small cliques have settled down in a fructuous digital location to perpetuate their physical activity. Encrypted messaging services enable terrorist groups to ‘transplant’ their entitative organizational chart onto digital platforms. Moreover, even if we present them separately for clarity’s sake, one must remember that they act in joint unison with their physical counterpart: they trigger an *onlife* radicalization process.

While radical groups’ activities on mainstream social media can be considered as providing broad radical milieus that aid novices’ initiation to violent Salafism, encrypted messaging services represent closed-circuit niches where hardliners can coordinate radicalizing operations in complete detachment from outgroup members (Shehabat and Mitew, 2018). As we will see, encryption contrasts with more popular platforms: it grants chatting apps with an “underground character,” and it makes them particularly resilient to infiltration attempts (Bloom et al., 2017). Our analysis focuses on the app Telegram, since research has pointed to this service as the fulcrum where ISIS cyberactivists mainly rearrange their maneuvers (Yayla and Speckhard, 2017). Telegram is a free multi-platform app that guarantees secure text exchange. It was launched in August 2013 by Nikolai and Pavel Durov, the creators of VKontakte – often known as the Russian Facebook. Telegram relies on channels and chatrooms. Channels are unidirectional structures where content is posted by centralized operators, and users cannot actively comment on them. Conversely, chatrooms are more dynamical and action-oriented. They enable (groups of) users to disseminate videos, radio broadcasts, memes, and other products (Shehabat et al., 2017). Importantly, they also signal the first step toward enclosed groups of like-minded individuals. Unlike major social networks, individuals’ entrances to such chatrooms require specific invitations. In this instance, these invitations are often links that are distributed directly by ISIS administrators. Moreover, most links are time-limited; they become inactive after a predetermined period (Bloom et al., 2017). This filtering mechanism allows ISIS Telegram officials to have a high selection control over the populace of such chatrooms so as to form radical conclaves. Another feature that augments the homogeneous degree of these environments concerns the relative facility to detect assorted sorts of interlopers – be it academics, journalists, or surveillance agents. Indeed, chatrooms’ registration of lists of active members, and the time of the latest posted content, allows ISIS supporters to ban so-called lurkers (inactive participants). This is exactly what happened to the authors of a 2017 study who were blocked on multiple chatrooms after extended periods of inaction (Bloom et al., 2017). These algorithmically regulated chatrooms ensure the “online killing” of the enemy, and they provide members with a clear-cut in-group space in turn. Put differently, an action-oriented involvement on the user part, who must show her loyalty to the Islamic State through a constant interaction lest cybersoldiers expel her from the group, promotes radicalization in such digital locations.

However, Telegram’s well-advertised algorithmic end-to-end encryption underlies the “underground” genesis of radical small cliques. Telegram applies this secrecy trait to one-to-one interactions, but research suggests that new protocols might extend this feature to the whole triad of new media communication. End-to-end encryption means that, during peer-to-peer communication, all data is exchanged only between the sender and the receiver (Shehabat et al., 2017). There is already some proof that shows perpetrators’ capacities to engage other trusted individuals in secluded and secret chatrooms (Meichtry and Schechner, 2016). In addition, all the messages can be automatically erased using a “self-destruct option” as soon as they reach their intended audience. Such a function places an investigative burden on intelligence agencies’ detection activities (Bloom et al., 2017). Accordingly, Telegram allows extremists to arrange themselves in entitative virtual cliques that mirror the structure of their physical counterparts. Enemies are kept at a distance and, if tracked, they get ousted promptly. On the other hand, secluded digital proximity promotes affiliative ties among ISIS sympathizers. Crucial, though, is the cloud-based nature of Telegram, which buttresses the *onlife* trend of the radicalization process. Versatile multi-platform entry points, admittedly, engender the possibility for radicalized individuals to cut symbiotically across physical and digital borders.

## Recruiting Through Videos: Immersive and Reality-Like Effect in Isis Propaganda

We have just seen the effectiveness of Telegram: it cuts across the dynamic boundary between online and offline radicalization experiences. However, in the Web 2.0 culture, even more traditional media solutions – like short amateur videos – have become a powerful tool of *onlife* experience. In particular, in ISIS’ recruitment activity, online videos are not a secondary tool; they are one of the trademarks that foster the radicalization of foreign fighters on a worldwide scale (Sardarnia and Safizadeh, 2017). Therefore, we will examine the textual and technical strategies that are employed in some of these media outputs. Specifically, we focus on the narrative and figurative features of the “actors”, on the camera use, and on the setting type^3^.

Our corpus comprises of six videos that were produced by *al-Hayat Media Centre* between 2014 and 2018. Three videos belong to the “Inside the Caliphate” format; they show foreign fighters’ description of their first-hand experience as soldiers of the Islamic State. The remaining videos are *nasheeds*; these files visually explain the urgency to join jihad, the life in Syria, and the treatment of the enemy. Different factors dictated our choice. First, the corpus offers a thorough picture of different *onlife* components that drive the radicalization process. Second, *al-Hayat* is the official media wing of the Islamic State, and it is involved in the production of contents that are aimed at Western audiences and recruits (our primary source of interest) (Milton, 2018). Furthermore, our analysis of videos released only by *al-Hayat* made sure that the footage was officially ISIS-branded, and that such footage was consistent in its technical features. We provide the complete list of videos at the end of this paper. We accessed all the videos on https://jihadology.net/.

As we have already said in the introduction, we are well aware of our non-statistical approach and the quantitative limits of the corpus used. Nevertheless, we think the corpus fits a textual analysis concerned with pinpointing the *onlife* character inscribed in ISIS’ radicalization. In fact, in textual qualitative analysis what matters is the significance of the corpus not its representativeness on a statistical base. Just as in ethnographic analysis, the analyst aims to highlight a general anthropological problem through the focus on a very specific case.

To begin with, our analysis considers the *us* vs. *them* opposition – mentioned in section three – as a structural feature that establishes a recurrent pattern throughout the corpus. On the one hand, the *us* of the Islamic world is always depicted as plural and manifold: it portrays a world that is inhabited by many people, recognizable faces, and multiple thematic roles. These roles include soldiers, religious leaders, and fathers. Additionally, all of these characters build on an atmosphere of togetherness that is reminiscent of the *ummah* that ISIS tries to create. Physical proximity (people hugging) and coordinated movements (groups in circles around a flag) constitute this community sense. Above all, though, foreign fighters’ testimonies, coupled with a massive use of close-ups, establishes the Islamic world’s personalization: in short, ISIS’s world is a human world. On the other hand, the *them* of the Western world is undifferentiated and depersonalized. Accordingly, enemies, such as the USA and the Soviet Union, are lumped together. Christians and Jews become interchangeable entities. Moreover, a simplistic narrative frame reduces the Western world either to its deceiving leaders or to graphs. In other words, it is as if, in the sphere of *them*, there is no “real” life and humanity. Rather, there are only powerful lying leaders. This true-false rhetoric crosses the entire corpus. Islam is not only a true doctrine; it is also an *authentic* world that is made up of active ordinary people that continuously unfold on the screen. Conversely, the West is false and corrupt – both in its values, and in its idols/leaders. In these videos, ISIS generates an Islamic humanization through their differentiation in subjectivities’ constructions. Indeed, even before the ascription of explicit and positive values to Muslims themselves, life and humanness are inherently present within them. For one thing, these videos manipulate their addressees, and they call them to action in an explicit way. Additionally, they offer such a delegitimized and depreciated representation of the enemy to make “natural,” and spontaneous, any type of aggressive reaction against it.

The interrelation between realism, and the immersive strategy^4^ that feeds – in a certain way –radicalization’s *onlife* dimension is an even more interesting factor in this corpus. As we have already said, the *onlife realm* characterizes the lives of us all, but it is particularly salient in the media productions that ISIS broadcasts online. ISIS’ videos always adopt a realistic style where the viewer is not presented with a distant Islamic world. Contrarily, the viewer gets immersed in what we call a *reality bath*. This reality bath seems to take on two different declinations in the corpus: a *testimonial declination* (1) and a *video-ludic declination* (2) that, however, is never “unrealistic.” The *testimonial declination* emerges in the videos that have a strong protagonist: a militant who explains the reasons, and the meaning, of the battle to be undertaken. The militant often shows, and quotes, the Koran. More generally, he cites the values of jihad and the importance of setting an example to others. Moreover, in order to strengthen the credibility of his battle experience, the militant’s body sometimes shows the uncensored effects of the war. For example, in “Islamic State: Inside the Caliphate 6,” the solider is seriously injured or irreparably crippled. Yet, his disabled condition – which exonerates him from further combat – testifies his greatness and resilience, while it implicitly invites all of those who enjoy a better physical shape to take action. Indeed, such individuals have no excuse to stay inoperative. Likewise, these videos’ concreteness is another crucial aspect of such testimonies. Importantly, they are not abstract lessons in jihadist Islamic doctrine, and they are not *fatwa* interpretations. Rather, they are credible testimonies that are *authenticated* by flesh-and-bone individuals with first and last names. Relatedly, the protagonists of videos like “Islamic State: Inside the Caliphate 2,” and “Islamic State: Inside the Caliphate 6,” are Abu Adam – from Australia – and Abu Salih from America. These two very recognizable figures introduce their war experience by directly addressing the viewer, and they also invite the viewer to take action against the enemy. This call to arms addresses those who are either at home or in Syria. Such an invitation is very detailed, and it often comprises a list of weapons, or strategies, to carry out a successful terrorist attack. In so doing, the two fighters establish a very intimate relation with the viewer who eventually may “exit” the online video and contact them, or who may attempt to take action herself. In other words, Abu Adam and Abu Salih represent the enlisters who bridge the gap between the battlefield and the bedroom with their online solicitation, and they possibly produce offline effects in a typical *onlife* circuit (Winter, 2015)^5^.

Another noteworthy feature is how the realistic scenario, in which these witnesses are immersed, is presented as their “natural environment.” Most of the time, the action takes place in a Middle Eastern post-war landscape. This landscape is marked by desert ground and semi-destroyed buildings. Accordingly, the camera captures the scenery where the battle took place and where the potential recruits may find themselves fighting. Interestingly, the presence of a glorious *mujahidin* in a desolated landscape made of ruins establishes a sort of pattern throughout various videos. Indeed, the *mujahadin*’s stature suggests that jihad – irrespective of where it takes place – entails these places. This is the case in “Islamic State: Inside the Caliphate 6” and “Islamic State: Inside the Caliphate 2.” In both examples, battleground realness’s emphasis marks an abrupt change from abstract room walls, or caves, that other terrorist groups prefer to use as venues for video recordings. While a realistic environment erases the specificity of the venue, it also associates militancy with a concrete scenario. In a certain sense, it accustoms the target audience to a future landscape of destruction. In this way, the viewer becomes the recipient of a double-realistic manipulation^6^ : a direct testimony that bears all the brutality of war on the fighters’ bodies signals these recordings’ *authenticity*, and a recurrent situation – detached from all abstract teachings – facilitates these videos’ *concreteness*.

Instead, the declination – which we call *video-ludic* – gives up the testimony’s force to strengthen the simulacrum of a *close experience* of war. It exposes the violent actions, the victims, the blood, and the bodies without hesitation in an extremely “raw” way that does not place any constraint on the contents’ ethical visibility. In videos such as “Answer the Call” or “Oh Disbelievers of the World,” ISIS media operators construct this uncensored visibility by using close shots and very fast editing. This option provokes an action-oriented *effect of accumulation* where concrete battle snippets are amassed together without a precise order. Dozens of decapitations, and blood-soaked knives, give the viewer an uncut image of what ISIS jihad is all about. The videos’ editing speed is reminiscent of the fictional video-ludic dimension that is typical of first-person shooters and Hollywood blockbusters. They also possess an underlying emotional pattern that involves the Western spectator through disgust and excitement. However, the same fast-paced editing allows one to *expand the real*, and it allows one to multiply it in its various facets. In so doing, this technique submits the viewer in a few minutes (3 or 4 on average) to a rich range of “concrete cases” that, again, create the *immersion effect*. To put it bluntly, the multiple close-ups of throat-cutting and dismembered bodies evoke a near-pornographic characterization, which inscribes the plain emotional aspect in reality’s crudeness.

In these videos, a combination of narrative strategies, such as the camera closeness and the footage speed, act out the aforementioned “bath of reality.” These strategies do not allow the eye to linger on any detail; instead, they overwhelm the viewer with all the weight, and the violence, of plain reality. Unlike Hollywood films, these videos portray the death of *real* human beings – even if the fast editing somehow mitigates this effect. In these videos, ISIS media productions do not leave anything undefined. Potential recruits are seduced with *the proof of a concrete experience* – whether painful or violent – that is testified by injured, but resilient, *mujahidin* of which the recruit aims to become a mirror image. Relatedly, the word “example” recurs several times in these videos. On-the-ground jihadists are, and must set, an example for both close radicalizing peers, and they are also examples for distant vulnerable enemies.

Media productions’ depictions of normality is another interesting, and perhaps counterintuitive, feature that ISIS uses to draw its online recruits to Syria. In videos such as “Islamic State: Inside the Caliphate 5” or “Our State is Victorious,” we find no “exceptional man,” no abstract model, and no special life. Gestures and moments are common and everyday. For example, daily scenes where children run through the streets, and where ordinary men talk and clean their weapons, are shot with fixed – or disorderly moving – cameras, create an *effect* of amateurism that aims to convey spontaneity, authenticity, and sincere initiative impulse. Ultimately, potential online members see neither an abstract recruitment protocol nor a spectacle of exceptions. Rather, they take a reality bath in their future offline world.

## Conclusion

Radicalization is a complex phenomenon. New technologies, and especially the internet, affect the violent trajectories of different terrorist offenders more and more frequently. However, building on previous research, our study suggests that, when one considers internet-based radicalization, the sphere of digital engagements should not be treated as separate from physical interactions. Rather, radicalization processes evolve, and develop, by integrating elements that pertain to both. This happens, for example, in the construction of online social environments. Here, the interaction between users and algorithm-based platforms structures radicalization-oriented echo chambers by incorporating users’ online, and offline, information simultaneously. Consequently, we argue that radicalization should be seen to take place in *onlife* echo systems: hybrid locations where users’ online interactions are partially determined by their everyday physical behavior and vice versa. Dataveillance and portable devices, in fact, establish a 24/7 cycle where radicalization can dynamically unfold on Facebook pages and in private houses. The awareness of online communication’s integration with offline experience has led ISIS to exploit the radicalization potential of the *onlife* dimension. Such exploitation has occurred on mainstream social media and on Telegram. In these hybrid echo-systems, potential recruits can radicalize with the help of like-minded peers and consensual media products. Relatedly, ISIS has been very keen to design compelling videos: these videos connect the viewer directly to on-the-ground foreign fighters. The latter provide a concrete testimony of jihadi life; they close the gap between the bedroom and the battlefield, and they can tip recruits over the edge in turn. Here we should not think of any media-reality determinism. Instead, we should consider how the projective media potential of these heroic figures, which explicitly invite one to follow their lead, fosters radicalization. To highlight this pressing aspect, we have adopted a textual approach that investigated how ISIS has leveraged on the soldier-viewer *onlife* relationship to build a communicative dimension where manipulation and identification are the cornerstones that push potential recruits toward violence. With regard to this point, we think that the combination of existing and new narrative approaches represents a fruitful way to better design counter-radicalization programs by exposing ever-evolving *onlife* features of violent extremism.

The present study represents a first step toward a reframing of radicalization as a complex *onlife* process that surely needs further elaboration. Indeed, our ISIS-centered analysis presents some peculiarities that may limit its extension to other radicalization contexts. On the one hand, ISIS has been the only terrorist actor, so far, that has coupled the efficacy of its online presence with the administration of a physical Caliphate. The possibility of branding its *onlife* character around the broadcast of exciting war footage and day-to-day governance might lie at the basis of ISIS’ success and is out of reach for other extremist organizations with different political or religious agendas. On the other hand, our case studies and text-based methodology have no statistical relevance and do not offer explicit guidelines for a systematic implementation in current counter-radicalization programs. This is why we encourage follow-up content analysis and longitudinal studies on extremists’ online and offline behavior to complement this rather new approach. Furthermore, we advocate an interdisciplinary effort aimed at distinguishing between the various algorithm types that regulate users’ activities on different platforms; this is an essential step if any counter-radicalization intervention is to be successful. Nonetheless, despite recognizing its restrictions and within the limits of available empirical data, our research has shown the intertwinement between the online and offline realms in today’s violent extremism. Most importantly, it has laid the basis for new approaches to update current intervention strategies. As regard to this point, we would like to provide some recommendations for the development of future de-radicalization programs that take the *onlife* character of radicalization into account: (1) one-sided measures that exclude either the offline or the online side of radicalization should be avoided – the development of grids to evaluate whether both realms have been considered may be a helpful technique; (2) we highly encourage violent extremism scholars to incorporate and closely monitor the findings and methods employed in related research branches around the relationship between digital interactions and offline behavior and vice versa; as a matter of fact, the tight effects of internet participation on physical activities among mainstream population is well-established among internet study experts; adapted to violent extremism such effects could provide new insights to be included in counter-radicalization efforts; (3) to better grasp the ways in which online and offline components intertwine in the process of radicalization, governments and organizations should partner up with private social media companies and demand for explanatory tools that account for the local layout of a user’s newsfeed. In short, platforms should provide clear reasons as to why their algorithms are presenting users with those specific contents and friends’ choices (Reed et al., 2019); (4) participants involved in de-radicalization programs and their friends could take part in experiments of content selection on social media. Coupled with follow-up self-reports, these experiments could shed new light on the interrelation between individuals and algorithms in the radicalization process. However, we recognize that a clear picture of dynamics that are involved in *onlife* locations is a difficult task, particularly in the case of an “underground process” like radicalization. *Onlife* environments change by the hour: algorithmic data-gathering activity constantly updates an ever-increasing user’s datafied image. In other words, what regulates my *onlife* echo-system today may be different from what regulates my *onlife* system tomorrow.

## Author Contributions

DV, AL, and AS contributed to build the hypotheses that underlie this study. DV wrote the article, except the chapter “Recruiting through Videos”, which was written by AL, and the conclusion, which was written by DV and AL. DV, AL, and AS together were in charge of subsequent revisions.

## Conflict of Interest

The authors declare that the research was conducted in the absence of any commercial or financial relationships that could be construed as a potential conflict of interest.

## Footnotes

1 The expression Web 2.0 signals a shift in the Web structure toward online social environments where users can actively participate through the creation and dissemination of contents on multiple platforms (Beer, 2009).

2 As a matter of fact we consider radicalization to be the results of recursive individual-environment interactions.

3 Our textual-semiotic approach is based on Greimas (1970). The basic idea of the semiotic approach is that the internal narrative organization of each text (be it verbal or visual), creates meaning effects and these meaning effects shape our minds, our behavior, our emotions, so that they strongly condition our experience in the world. The narrative level of texts does not merely consist of the explicit elements making up the narration, but it also include the implicit developments, the implicit values, and the implicit connotations associated to some given features (i.e., if a text recurs to the image of a battle, this implies a narrative pattern made of enemies, weapons, strategy, decisional hierarchy, and so on). An in-depth look at the narrative level enables the analyst to grasp some implicit semantic elements which condition the viewer’s behavior without being explicit said. This narrative approach is crucial for the whole semiotic approach to cultural dynamics (as in Lorusso, 2015).

4 We use *immersive strategy* in the way it is intended in contemporary media studies (Lister et al., 2009; Rose, 2012). Rather than contents or stories, media today aim to produce experiences that entail the sensory stimulation of the viewer. Such sensory stimulation comprises the viewer’s projection, emotional involvement and identification. A paradigmatic example of this strategy are video games and all forms of augmented reality. A last element at the basis of immersive communication is what Jenkins (2006) calls *media convergence*, i.e., the interaction between different media that produces integrated consumption flows.

5 We cannot neglect the potential simulacrality of these enlisters. In fact, the whole recording could be fake or carefully performed – a sort of make-believe.

6 According to Greimas (1970), every text or media product entails, first and foremost, a manipulation of the addressee to make her believe what the text supports. Along these lines, *manipulation* is not a negative discursive aspect; it is rather, a necessary narrative phase that shows how there cannot be neutral or unbiased texts.
